# Treatment-resistant depression and intranasal esketamine: Spanish consensus on theoretical aspects

**DOI:** 10.3389/fpsyt.2025.1623659

**Published:** 2025-08-04

**Authors:** Fernando Mora, Jose Antoni Ramos-Quiroga, Enrique Baca-García, José Manuel Crespo, Luis Gutiérrez-Rojas, Aránzazu Madrazo, Lucía Pérez Costillas, Pilar A. Saiz, Vicente Tordera, Eduard Vieta

**Affiliations:** ^1^ Department of Psychiatry and Mental Health, Hospital Universitario Infanta Leonor, Madrid, Spain; ^2^ Department of Legal Medicine and Psychiatry, Complutense University, Madrid, Spain; ^3^ Department of Mental Health, Hospital Universitari Vall d’Hebron, CIBERSAM, VHIR, Universitat Autònoma de Barcelona, Barcelona, Spain; ^4^ Group of Psychiatry, Mental Health and Addictions, Vall d’Hebron Research Institute (VHIR), Barcelona, Spain; ^5^ Department of Psychiatry and Forensic Medicine, Universitat Autònoma de Barcelona, Barcelona, Spain; ^6^ Biomedical Network Research Centre on Mental Health (CIBERSAM), Barcelona, Spain; ^7^ Department of Psychiatry, Jimenez Diaz Foundation Hospital, Madrid, Spain; ^8^ Department of Psychiatry, Instituto de Investigación Sanitaria (IIS)-Jimenez Diaz Foundation, Madrid, Spain; ^9^ Biomedical Research in Mental Health Networking, CIBERSAM, Madrid, Spain; ^10^ Department of Psychiatry, Madrid Autonomous University, Madrid, Spain; ^11^ Department of Psychiatry, Rey Juan Carlos University Hospital, Mostoles, Spain; ^12^ Department of Psychiatry, General Hospital of Villalba, Madrid, Spain; ^13^ Department of Psychiatry, Infanta Elena University Hospital, Valdemoro, Spain; ^14^ Department of Psychiatry, Nimes University Hospital, Nimes, France; ^15^ Departmento of Psychiatry, Complejo Hospitalario de A Coruña, A Coruña, Spain; ^16^ Psychiatry Service, Hospital Universitario San Cecilio, Granada, Spain; ^17^ Department of Psychiatry and CTS-549 Research Group, Institute of Neurosciences, University of Granada, Granada, Spain; ^18^ Psychiatric Hospitalization Unit, Hospital Universitario de Basurto, Bilbao, Spain; ^19^ Mental Health Clinical Unit, University Regional Hospital of Málaga, Biomedical Research Institute of Malaga (IBIMA), Málaga, Spain; ^20^ Grupo de Investigación en Salud Mental (INTRAM) (PAIDI CTS456), Málaga, Spain; ^21^ Department of Public, Health and Psychiatry, University of Malaga, Faculty of Medicine, Málaga, Spain; ^22^ Department of Psychiatry, University of Oviedo, Centro de Investigación Biomédica en Red en Salud Mental (CIBERSAM), Instituto de Investigación Sanitaria del Principado de Asturias (ISPA), Oviedo, Spain; ^23^ Instituto Universitario de Neurociencias del Principado de Asturias (INEUROPA), Servicio de Salud del Principado de Asturias (SESPA), Oviedo, Spain; ^24^ Psychiatry Service, Hospital Lluis Alcanyis, Xativa, Spain; ^25^ Department of Medicine, Faculty of Medicine and Health Sciences, University of Barcelona (UB), Barcelona, Spain; ^26^ Bipolar and Depressive Disorders Unit, Hospital Clínic de Barcelona, Barcelona, Spain; ^27^ Institut d’Investigacions Biomèdiques August Pi i Sunyer (IDIBAPS), Barcelona, Spain; ^28^ Centro de Investigación Biomédica en Red de Salud Mental (CIBERSAM), Instituto de Salud Carlos III, Madrid, Spain

**Keywords:** intranasal esketamine, definition, therapeutic inertia, Spain, consensus

## Abstract

**Introduction:**

Depression is a highly prevalent disease that severely impacts the life of patients. Inadequate response to at least two antidepressants despite adequacy of treatment and adherence is known as treatment-resistant depression (TRD), which entails a higher social and economic burden than non-resistant major depression. The lack of consensus on the definition of TRD and other aspects complicates management of the disease. Intranasal esketamine has a novel mechanism of action that differs from that of traditional antidepressants by improving neuroplasticity and synaptogenesis.

**Material and methods:**

A scientific committee comprising ten psychiatrists, experts in TRD in Spain, reviewed the literature (grey literature and articles or scientific communications published between January 2014 and January 2024 in PubMed) and developed statements on theoretical and conceptual aspects of TRD. Statements were developed in a first meeting following a discussion group approach, refined in a second meeting following a nominal group technique, and consensus was finally drafted in a third meeting.

**Results:**

A series of statements and recommendations were developed. Definitions for TRD and clinical response were proposed. The impact of therapeutic inertia was highlighted, identifying its causes and consequences. The role of intranasal esketamine in the TRD therapeutic treatment landscape was reviewed, and a treatment algorithm was developed, including specifics on evaluation of response to avoid therapeutic inertia and ensure an adequate treatment.

**Conclusions:**

This is the first consensus developed in Spain regarding theoretical aspects of TRD and the role of intranasal esketamine in TRD therapeutic approach. A definition of TRD was proposed, together with a treatment algorithm.

## 1 Introduction

Major depressive disorder (MDD) has a lifetime prevalence of approximately 20%—more common among women than men—and its mood, physical, and cognitive symptoms include anhedonia, anxiety, distress, impaired functioning, and suicidal ideation (1, 2). Accordingly, depressive disorders are among the diseases that most impact quality of life and that lead to a major loss of disability-adjusted life years (3). Despite the availability of multiple treatments for MDD, not all patients respond or achieve remission with the first treatment (4, 5). Inadequate response to at least two antidepressants (AD) despite adequacy of treatment and adherence is known as treatment-resistant depression (TRD) and it can affect up to 55% of MDD patients (6–8). TRD presents a different clinical profile, comprising a higher social and economic burden than MDD derived from the poorer quality of life, reduced functioning, greater work impairment, and a higher suicidal ideation and hospitalisations. These specific features result in a greater use of health care resources (9–11). Furthermore, TRD has a longitudinal dimension, which is essential for comprehending the relapsing nature and the persistence of depressive symptoms over time. The main goal of treatment of MDD is to achieve clinical remission, also aiming to achieve the highest possible levels of functionality. Therapeutic strategies also focus on preventing relapses during the maintenance phase. However, the likelihood of clinical response and remission decreases with late response to treatment or with failure to successive treatments (5, 12–15). This underscores the importance of an early evaluation of response and clinical evolution to adequately identify patients who may develop TRD and optimise their treatment to improve prognosis. One of the challenges in identifying and treating patients with TRD is the variability of its definition. There is no internationally recognised consensus on what constitutes TRD or how different types of therapies (e.g. pharmacotherapy, psychotherapy, neurostimulation) should be considered, which leads to heterogeneous and inconsistent estimates of TRD incidence and prevalence, and results in varying management and outcomes that may not reflect the clinical and humanistic burden of the disease (6, 16, 17). The lack of consensus regarding TRD also includes the definition of therapeutic goals and response, and an evidence-based treatment algorithm.

Traditionally, pharmacological management of MDD consisted of AD that target the monoaminergic pathway; however, a clinical effect may not be evident for several weeks, and many patients struggle to resolve depressive symptoms during treatment (18). Because of this, other mechanisms of action have been explored and new therapies have been approved in recent years, widening the therapeutic arsenal (19). In this context, glutamate is the most abundant excitatory neurotransmitter in the brain and plays a key role in synaptic plasticity and the formation of new neuronal connections (20). Alterations in glutamatergic signalling have been consistently observed in patients with depression compared to healthy controls, supporting its involvement in mood regulation and neurocircuitry dysfunction. Since the early 2000s, approximately 20 glutamate-modulating compounds have been evaluated in human clinical trials, reflecting growing interest in this target (20). One of these new therapies is intranasal esketamine, which is one of the few non-monoaminergic-targeting treatments approved for depression; instead, it targets the glutamatergic pathway, improving neuronal plasticity and promoting synaptogenesis (18, 21). A comprehensive clinical development programme in patients with TRD demonstrated that intranasal esketamine combined with an oral AD led to a rapid improvement of depressive symptoms and delayed relapse compared with an oral AD (22). A head-to-head study showed a significant benefit of intranasal esketamine compared with a different augmentation strategy (23). Further, an indirect adjusted comparison of clinical trial and real-world data revealed intranasal esketamine was superior to currently used polypharmacy strategies (24). Notably, intranasal esketamine is the only AD approved specifically for TRD in Europe and is also the only AD in the European Medicines Agency’s (EMA) critical medicine list (25). Its approval has helped substantiate the relevance of glutamate in the pathophysiology of depression, particularly in treatment-resistant cases. These agents represent a promising new avenue for intervention, especially in patients who do not respond adequately to conventional monoaminergic drugs. The therapeutic potential of glutamate-based treatments lies not only in their distinct mechanism of action, but also in their capacity to exert faster and potentially more robust AD effects (18).

However, a standardised care plan for patients with TRD treated with intranasal esketamine is lacking in Spain. Uncertainty persists regarding the optimal timing, criteria for prescription, and availability of professionals experienced in managing patients with TRD and administering intranasal esketamine. Within this context, the present study aimed to address gaps where existing literature is unclear. We propose standardised definitions related to TRD, outline therapeutic objectives for these patients, and present a treatment algorithm involving intranasal esketamine to support routine clinical practice and potentially enhance patient outcomes.

Considering the challenges of identifying and managing patients with TRD, we aimed to provide guidance on topics on which the evidence was unclear. Here, we proposed a set of definitions related to TRD, clarified the therapeutic goals in TRD, and provided a treatment algorithm to improve patient outcomes, in our clinical opinion.

## 2 Materials and methods

### 2.1 Study design

A scientific committee comprising ten psychiatrists who were experts in TRD and practiced in Spain reviewed the literature and developed statements to find consensus on the definitions and theoretical aspects of TRD. The scientific committee discussed the statements in three online meetings to achieve consensus. First, a discussion session aimed to develop statements on the theoretical aspects related to the TRD. Then, a second meeting followed a semi-structured, nominal group technique, in which participants voted on the statements generated in the first session to achieve consensus. Finally, in a third meeting, the advisory committee reviewed the final conclusions and validated the consensus. Key points were then defined based on these results.

### 2.2 Literature review

A targeted literature review was conducted in PubMed to identify articles and scientific communications published between January 2014 and January 2024. The search strategy included the following terms: “treatment-resistant depression,” “TRD definition,” “partial response,” “therapeutic inertia,” “treatment algorithm,” “glutamate,” “esketamine,” and “rapid-acting antidepressants.” Inclusion criteria comprised original research articles, systematic reviews, clinical guidelines, and expert consensus documents published in English or Spanish, focused on adult patients with TRD. Additionally, grey literature was reviewed, with particular attention to documents available on the websites of medical associations and scientific societies relevant to TRD.

### 2.3 Development of statements

The results of the literature review were used to identify topics that were unclear or on which the evaluated sources differed. The first meeting followed a discussion group approach in April 2024 aimed at developing statements (26). Afterwards, a list of statements was shared with all the members of the scientific committee to assess the degree of agreement or need for modifications. The second meeting, in June 2024, followed a formal consensus development approach in the form of the nominal group technique, where the statements and recommendations were refined (27). This approach, which is routinely used in consensus reports, is particularly appropriate in contexts where there are few specialists in a particular field and helps to reach agreement. Using this procedure, the panel developed an extensive list of statements, including only those for which there was full consensus.

This methodology, commonly employed in consensus documents, is particularly suitable when the number of experts on a given topic is limited, facilitating consensus achievement (28, 29). Through this process, the panel elaborated a comprehensive list of statements, only including those that achieved unanimous consensus. The consensus achieved, and the conclusions derived from the second meeting were discussed by the scientific committee in a third and final online meeting. A report gathering the consensus reached was reviewed by the scientific committee to ensure the adequate statements and context had been provided. The final statements regarded four topics: TRD-related definitions; therapeutic consensus and partial response; therapeutic inertia in TRD; role of intranasal esketamine in the management of TRD.

The study was funded by Johnson & Johnson, who did not participate in the study design or interpretation of results.

## 3 Results

### 3.1 Topic 1. TRD-related definitions

#### 3.1.1 TRD definition

Consensus:

TRD was defined as depression that has failed to improve after receiving two treatments strategies that have different pharmacodynamic/receptor profiles.

Evidence/discussion:

There are more than seven published definitions of TRD (6). The definitions used by the EMA (30) and U.S. Food and Drug Administration (FDA) (31) concern AD and guide inclusion criteria in clinical trials; however, the scientific committee considered that these definitions do not align with management of TRD in clinical practice. We propose ‘treatment strategies’, as it is a wider term that encompasses pharmacological and non-pharmacological approaches (such as psychotherapy and neurostimulation, the latter excluded from the EMA definition) and better reflects the potential treatment options for TRD in clinical practice (30, 32). We also propose using ‘pharmacodynamic/receptor profile’ in the definition; although most available treatments target the monoaminergic pathway and, thus, have the same mechanism of action, different agents target specific receptors and transporters. TRD is different from MDD, which is associated with a strong functional decline and higher suicidal ideation and hospitalisations (9, 33).

#### 3.1.2 Depression resistant to monoamine transporter inhibitors

Consensus:

There is a need to define depression resistant to monoamine transporter inhibitors, currently lacking in the literature, to facilitate therapeutic management.

Evidence/discussion:

Traditional AD target the monoaminergic system (34, 35); however, not all patients respond to these strategies, which suggests that depression involves additional pathways beyond the monoaminergic one. ‘Depression resistant to monoamine transporter inhibitors’ is a concept that may be useful from a clinical practice perspective and in guiding therapeutic approaches. This concept refers to a clinical subtype of MDD characterised by poor response to traditional monoaminergic AD and associated with alterations in amino acid neurotransmission, particularly glutamate and GABA, rather than abnormalities in serotonin, norepinephrine, or dopamine systems. This clinical concept is supported by growing neuroimaging and neurochemical evidence indicating that patients with TRD often exhibit reduced levels of GABA in the anterior cingulate cortex (ACC), diminished glutamate/glutamine (Glx) concentrations, and decreased hippocampal volumes—findings that are not consistently observed in treatment-responsive patients or in healthy controls (36–38). The term ‘depression resistant to monoamine transporter inhibitors’ encapsulates a shift in the conceptual and clinical framework of TRD, emphasising the need for personalised treatment strategies based on underlying neurobiology rather than sequential monoaminergic pharmacotherapy alone. Further exploration of the concept of ‘depression resistant to monoamine transporter inhibitors’ is needed to adequately distinguish the specific treatments to which patients are resistant, rather than referring to treatment resistance in general.

#### 3.1.3 Neurobiological definition of TRD

**Figure 1:**
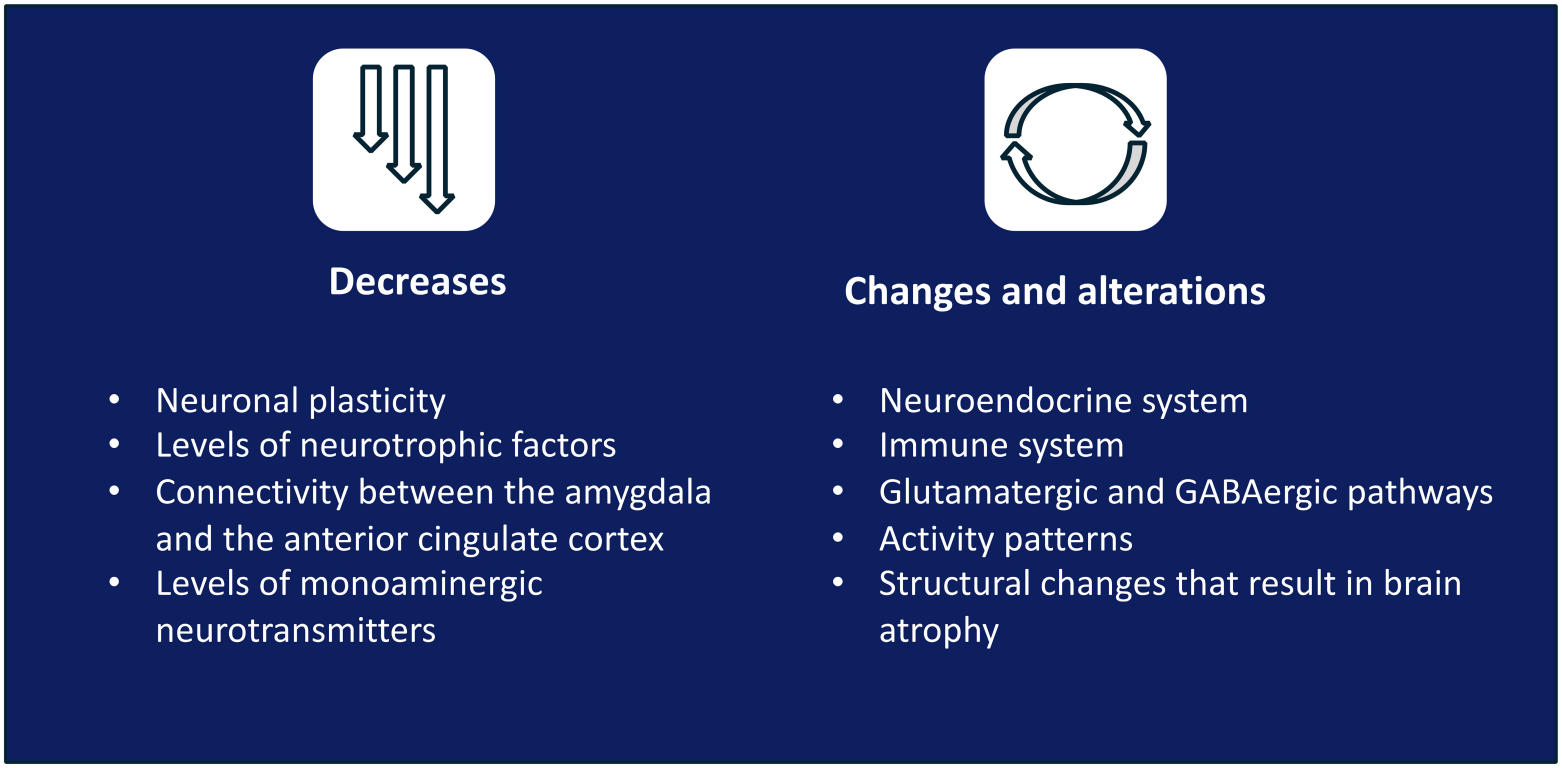
Neurobiological definition of TRD.

Consensus:

Depression causes neuroplastic, neuroendocrine, and neurotransmission changes (**Figure 1**). Depression leads to i) a decrease in neuronal plasticity and levels of neurotrophic factors, alteration of the connectivity between the amygdala and the anterior cingulate cortex, and an imbalance in monoaminergic neurotransmitters; ii) alterations in the neuroendocrine system, immune system, glutamatergic and GABAergic pathways (leading to an alteration of activity patterns), activity patterns, and structures (the latter resulting in brain atrophy).

Evidence/discussion:

TRD is multifactorial and is caused by biological, genetic, psychological, social, and family factors (35). The monoaminergic theory of depression does not fully explain the changes observed, such as the existence of lesions in the brain or damage in the neural architecture from the beginning of the disease (39, 40). Thus, there is a need for better characterisation of the neurobiological alterations that occur in depression and promote further changes to, ultimately, improve treatment. In this framework, neuroprogressive mechanisms, including oxidative stress, mitochondrial dysfunction, inflammation, and neuroendocrine imbalance, have been associated with the cumulative neurobiological burden observed in depression and with increased risk of relapse and treatment resistance (41).

Alterations in the concentration of neurotrophic factors (brain-derived neurotrophic factor [BDNF] being the most studied) during depression result in altered functional plasticity, neuronal atrophy, and reduced synaptic connectivity and function (38, 42, 43). The correct signalling of BDNF is crucial for preserving the emotional, cognitive and behavioural response of the brain to a changing environment, a flexibility that is compromised in people with depression (44). Furthermore, the clinical performance of AD has been proposed to rely on their effects on neurogenesis, neuronal structure, and synaptic activity (45) and, therefore, the difference in clinical performance of different agents would be supported by their effectiveness in restoring neuroplasticity (46). The neurotransmitters most heavily studied in TRD are monoamines, glutamate, and GABA. Low levels of monoamines (primarily serotonin, dopamine, and norepinephrine) detected in patients with depression have been associated with emotional regulation (47). This neurochemical imbalance coexists with and is related to a reduction of neuroplasticity in depression (48). Levels of other neurotransmitters, such as glutamate and GABA, are also affected in depression. Specifically, the disruption of the glutamatergic pathway has been related to elevated levels of cortisol, malfunctioning of the immune system, and BDNF signalling and release. Therefore, the alteration of the glutamatergic pathway promotes and sustains, both directly and indirectly, brain atrophy (39, 41) Moreover, low GABA levels are observed in symptomatic patients but not in those with remission (49, 50). Alterations of the brain’s structure (including reduced volume of the hippocampus (42) and prefrontal and cingulate cortex (35)) and of the connectivity between brain regions are responsible for emotional and cognitive regulation during depression (51). Therefore, recognising neuroplasticity as a therapeutic target in depression, and TRD specifically, is essential.

#### 3.1.4 TRD in clinical practice

Consensus:

A patient with TRD does not experience sufficient improvement in their symptoms despite adherence to at least two treatment strategies that have different pharmacodynamic/receptor profile and that are given consecutively, following adequate timing and dosing. The following characteristics may help in identifying patients with TRD: i) insufficient improvement of symptoms with the first and consecutive treatments; ii) long or recurrent depressive episodes; iii) early relapse during treatment; iv) relapse after completing treatment; v) presence of residual symptoms.

Evidence/discussion:

It is estimated that approximately half of patients with MDD are not adequately diagnosed (52), which suggests that identifying patients with TRD is a major challenge for healthcare professionals. Notably, nearly a quarter of patients with bipolar disorder are initially misdiagnosed as having MDD, and this misdiagnosis often persists until a manic or hypomanic episode becomes clinically evident. This diagnostic overlap may lead to ineffective treatment strategies and contribute to apparent treatment resistance (53). On this note, knowledge of a patient’s complete medical history is key, as treatment approaches previously used by a patient can guide selection of the next treatment (32). Adherence to treatment is a factor to consider, given that lack of adherence is associated with worse outcomes and more adverse events (54–57). In Spain, 30–50% of patients are suspected to have poor adherence to AD (54–56). Adequate timing and dosing are included in the definition of a patient with TRD; there is no international consensus on this matter, but most studies consider response to treatment should be evaluated after 4–6 weeks at the optimal dose (6, 58). Evolution of each patient must be assessed according to the judgement and experience of the healthcare professional (HCP), as well as considering patient-reported outcomes (58), using validated tools such as the Montgomery-Åsberg Depression Rating scale, Hamilton Depression Rating Scale, or the EuroQol visual analogue scale.

#### 3.1.5 Therapeutic goals in TRD

**Figure 2:**
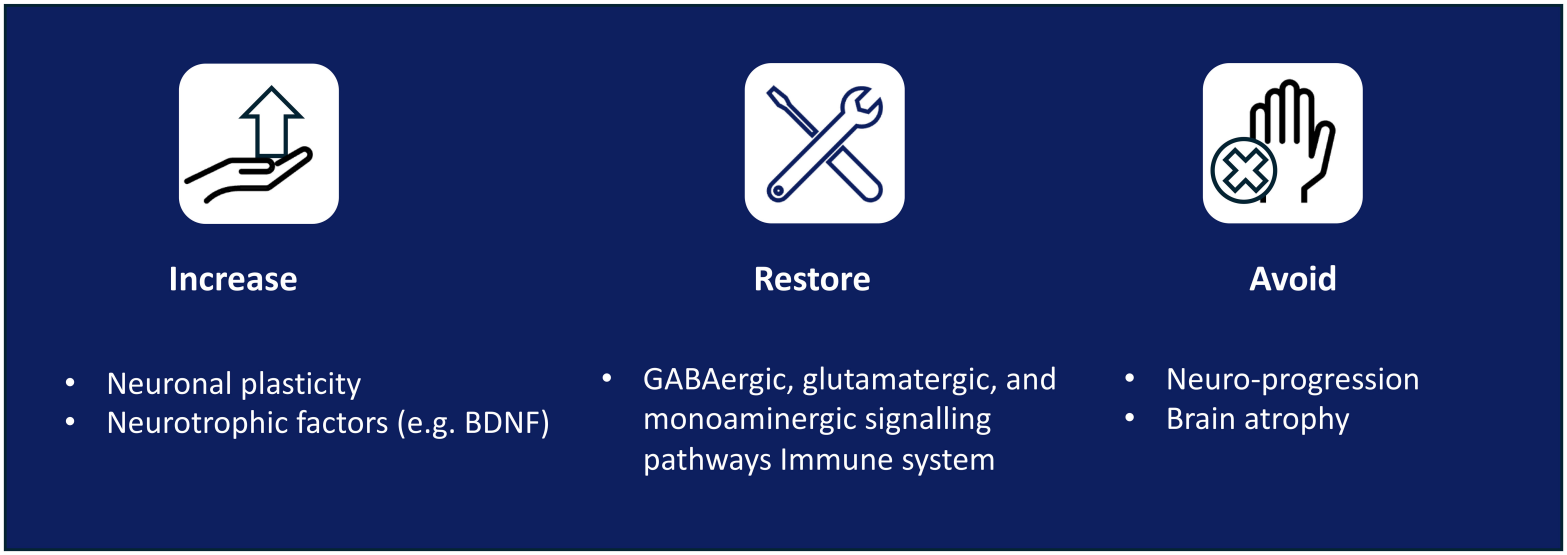
Therapeutic goals. BDNF, brain-derived neurotrophic factor.

Consensus:

Remission of symptoms and functional recovery should be achieved whenever possible. Given the neurobiological basis of depression, pharmacologic treatment should address neurobiological factors and consider the patient’s profile, although a wider therapeutic management is convenient. Therapeutic goals should be to: increase neuronal plasticity and neurotrophic factors; restore GABAergic, glutamatergic, and monoaminergic signalling pathways; and avoid neuroprogression and brain atrophy (**Figure 2**).

Evidence/discussion:

Remission of symptoms and functional recovery have been proposed as goals in the treatment of depression (16, 32). Depression is associated with alterations in the concentration of GABA and glutamate (39) and a loss of plasticity and brain connectivity, which impact cognitive and emotional regulation (44, 59–61). An improvement in depressive symptoms has been associated with an increase in BDNF levels (46, 61). Changes in neuroplasticity and improved neurotrophic support underlie effective treatment of depression (39, 62). Management of depression should include strategies beyond monoaminergic-targeting treatments, addressing neurobiological factors, and exerting an effect on different targets to promote neurogenesis and dendritic arborisation, and to increase synaptic connections to help revert brain atrophy and improve symptoms (39).

### 3.2 Topic 2. Therapeutic consensus and partial response

#### 3.2.1 Lack of response or partial response

Consensus:

Lack of response is defined as the total absence of improvement to treatment or a minor improvement that does not reach the minimum therapeutic goals during the evaluated timeframe.

Partial response is defined as a noticeable but insufficient improvement to treatment.

Lack of response and partial response require action to avoid prolonging an undesired health status and reduce neuroprogression and its consequences.

Evidence/discussion:

There are no globally accepted definitions for lack of response and partial response (31). However, a recent consensus document defined lack of response as a reduction in symptom severity <50%, and defined partial response as achievement of a >25% and <50% reduction in symptom severity (63). EMA released in 2024 a draft guideline that proposed the same definition of partial response (64).

#### 3.2.2 Therapeutic consensus

Consensus:

Clinical recommendations on management of TRD are heterogeneous. There is a need for: i) studies that reflect clinical practice; ii) head-to-head and comparative studies; and iii) algorithms that consider the most up-to-date evidence to standardise clinical recommendations.

Evidence/discussion:

The lack of homogeneity in treatment recommendations complicates management of TRD. There are few head-to-head studies, meta-analyses, and comparative studies of the available treatments (6, 65). There is also limited evidence of studies evaluating patients with TRD specifically. In this context, the comprehensive clinical programme of intranasal esketamine was developed considering patients with TRD.

### 3.3 Topic 3. Therapeutic inertia

#### 3.3.1 Causes of therapeutic inertia

Consensus:

The main cause of therapeutic inertia is the expectation from HCPs for a patient with a partial response to improve over time without modifying their current treatment.

**Figure 3:**
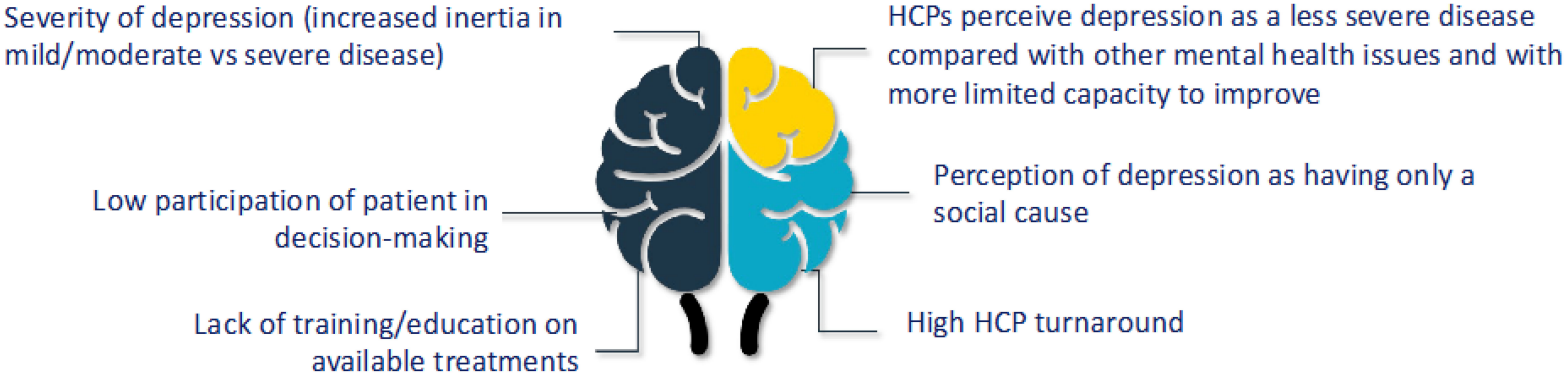
Secondary causes of therapeutic inertia.

Secondary factors that contribute to therapeutic inertia are: i) severity of depression (increased inertia in mild/moderate vs severe disease); ii) low participation of patient in decision-making; iii) lack of training/education on available treatments; iv) HCPs perceive depression as a less severe disease compared with other mental health issues and with more limited capacity to improve; v) perception of depression as having only a social cause; vi) high HCP turnaround (**Figure 3**).

Evidence/discussion:

Treatment inertia is the lack of treatment modification when the therapeutic goals have not been met. This concept is used in other diseases, such as diabetes, but it is relatively new in depression (66–68). Real-world studies revealed that patients with MDD often initiate treatment late (69) or are exposed to treatment inertia (approximately 30%) (66), the latter influenced by the severity of the disease and comorbidities (anxiety, hypertension). Psychiatric treatments are frequently maintained under the expectation that the patient will improve (70). However, delaying use of a treatment that achieves an inadequate response leads to worse clinical outcomes (71). Socio-demographic factors may predispose patients to TRD (4, 6, 72), and patients may also hide their symptoms from their treating HCP (61). Patients with TRD often feel hopeless, which impacts treatment adherence (32). We believe that HCPs perceive patients with TRD as less severe than those with other psychiatric conditions; however, there is no clinical or economic rationale for this (73). Indeed, evidence shows that TRD severely impacts the quality of life of patients (9, 74), leads to work and activity impairment (9), involves increased healthcare resource utilisation than MDD (10), and has longer unresolved episodes than MDD (74). Shared decision-making between the patient and HCP has been proposed as an approach that improves results, contributes to commitment to treatment for depression, and avoids therapeutic inertia (32, 75). High HCP turnaround, lack of coordination across specialties, and scarce training on new treatments challenge treatment of TRD (76).

#### 3.3.2 Consequences of therapeutic inertia in TRD

Consensus:

The main consequence of therapeutic inertia is worsening of clinical prognosis, which includes: i) decreased probability of symptom remission and functional recovery; ii) increased probability of relapse or new episodes; iii) resistance to treatment and chronicity of depression; iv) loss of confidence in treatments and psychiatry; v) higher clinical/therapeutic effort to achieve patient recovery; vi) higher work absenteeism and increased expenditure caused by the disease; vii) increased negative impact on quality of life.

Evidence/discussion:

The longer the delay until adequate treatment, the lower the remission rate (13–15). Longer duration of untreated disease has been associated with lower BDNF concentration and a reduced hippocampal volume (77, 78). These structural changes worsen depression and favour resistance to treatment (77). Moreover, a study conducted in the US found that patients who had received timely treatment had fewer hospital visits and incurred lower treatment costs than patients who had suffered therapeutic inertia (69).

The scientific committee scored the impact of therapeutic inertia on TRD on a scale of 1 to 5; the average score obtained was 4.13.

#### 3.3.3 Use of conventional antidepressants

Consensus:

Patients should be evaluated 2 weeks after initiating treatment to assess preliminary response to treatment as well as safety and tolerability. Response should be evaluated again 2–4 weeks after the preliminary assessment (4–6 weeks after initiating treatment). If there is a partial response and good tolerability, treatment should be continued and possibly combined with another treatment strategy; If there is a lack of response, a different AD should be used.

Evidence/discussion:

The consensus statement developed by McAllister et al. suggested continuing treatment and combining it with other treatment strategy if a partial response is detected (32). The benefit of changing treatment over continuing and/or combining it with another treatment is unclear (6).

#### 3.3.4 Benefits of early intervention

Consensus:

Benefits of early intervention include an improvement in neuronal plasticity, functionality, therapeutic results, and neurostructural alterations caused by the disease, and a reduction in the risk of suicidal ideation, chronicity of depression, and negative impact on the quality of life of patients and caregivers.

Evidence/discussion:

Early optimisation of treatment leads to better results in the short term, improves the probability of achieving complete functional recovery, and can reduce damage in the brain (4, 79–83). The STAR*D study found that early remission reduced the duration of symptoms of depression and also favoured functional recovery of patients (12, 84).

### 3.4 Topic 4. Treatment algorithm and role of intranasal esketamine

**Figure 4:**
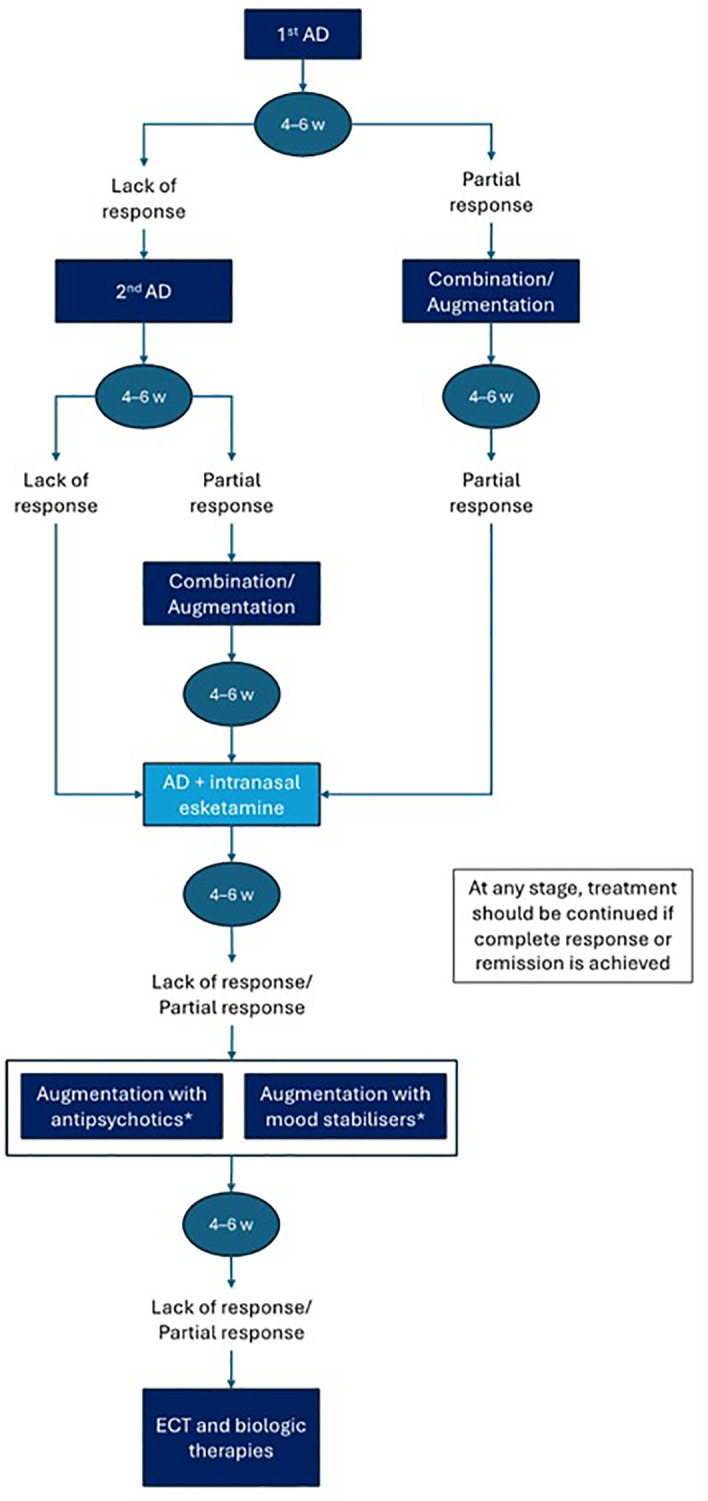
Treatment algorithm. AD, antidepressant; ECT, electroconvulsive therapy; w, weeks. *The choice between antipsychotics or mood stabilisers will depend on the patient's characteristics. Biologic therapies include yagus nerve stimulation and deep brain stimulation.

Consensus:

The committee developed a treatment algorithm for TRD, where intranasal esketamine would be used after not responding to two therapeutic strategies (**Figure 4**). This algorithm provides a structured, stepwise approach to TRD management, based on clinical evidence and expert consensus, and positions intranasal esketamine at an appropriate point in the therapeutic sequence.

Patients with major depressive disorder who do not achieve full remission after a first adequate AD trial (typically 4–6 weeks) may present with partial or no response. In cases of partial response, combination or augmentation strategies may be implemented—these may include the addition of mood stabilisers, atypical antipsychotics, or other pharmacological agents. If improvement remains insufficient, esketamine may be considered in combination with an oral AD. On the other hand, in patients with no response to a first AD, switching to a second AD from a different pharmacological class is recommended. If, after a second adequate trial the patient still does not respond, the condition is considered TRD. At this point, intranasal esketamine should be introduced in combination with an oral AD.

Following 4–6 weeks of treatment with AD plus esketamine, if there is insufficient clinical response, further augmentation strategies (e.g., with antipsychotics or mood stabilisers) may be considered, particularly in patients with mixed or bipolar spectrum features. In cases of persistent resistance, referral for electroconvulsive therapy or consideration of other biological treatments, such as repetitive transcranial magnetic stimulation, is recommended.

The main differences between intranasal esketamine and other AD are: i) it is the only AD specifically approved in Europe for TRD; ii) the mechanism of action differs from that of traditional AD and improves neuroplasticity and synaptogenesis; iii) faster onset of action; iv) tolerability is more predictable, since adverse events occur generally during the administration and observation period; and v) it is the only AD in the European Medicines Agency’s critical medicine list.

Evidence/discussion:

There is no consensus on when to evaluate response to treatment, but studies suggest that 4–6 weeks is an adequate timeframe (6, 58). Treatments must be optimised according to response, tolerability, and the patient’s profile (69). The use of intranasal esketamine is more restricted in Spain compared with the approved indication by the EMA, as it is only covered by the National Healthcare System in combination with selective serotonin reuptake inhibitors or serotonin and norepinephrine reuptake inhibitors in adults with TRD who have not responded to at least three AD strategies, one of them being a combination or an augmentation strategy. However, experts believe that intranasal esketamine should be used as soon as TRD is detected, given its proven efficacy and safety (6, 23, 58, 85–88). Furthermore, its inclusion in the EMA’s critical medicines list underscores its clinical relevance and therapeutic value (25).

## 4 Discussion

TRD is associated with poorer quality of life and more severely impaired functioning than MDD; despite multiple treatment options, remission is unattainable in many cases, which results in chronicity of depression and a marked neurobiological and neurochemical impact. Proper management of TRD requires adequate identification and early diagnosis (83). However, although TRD is generally understood as a lack of response to at least two lines of therapy (33), there is currently no globally accepted definition. In this study, we provided an overview of current theoretical aspects related to TRD and proposed a definition that considers treatments with different receptor profile and encompasses non-pharmacological approaches. We also reviewed the unmet needs in the management of this disease and made recommendations that aim to facilitate diagnosis and treatment of patients with TRD. Several consensus reports on TRD have been published in recent years, focused on management in particular countries or regions (89–91), or specific uses of TRD, such as its definition for clinical trials (63). Here, we have presented the first consensus report related to TRD focused on the context in Spain.

We highlighted therapeutic inertia as a crucial barrier to providing adequate treatment for patients with TRD, and we identified its main contributing factors and consequences. Treatment initiation for MDD is suboptimal (92), and delay in treatment initiation has been associated with worse outcomes (93). Moreover, the likelihood of remission of MDD decreases with late responses and increasing number of failed treatments ^(^ 5, 12–15 ^).^ Thus, it is of utmost importance to initiate treatment rapidly and closely monitor response to change management of the disease, if appropriate. Given the wide range of available treatment options, it is key to know a patient’s complete medical history to consider not only the number of previous treatments but also the type of prior failed lines, as they will both inform changes in management (16). Continuity of care plays a role in this and, for example, a study in Portugal revealed that only approximately half of patients with TRD continued follow-up with the same HCP who initiated treatment (94). Additionally, real-world studies reveal that length of follow-up varies widely (95), which may prevent proper assessment of response and also limit obtaining insights into factors that impact response.

This study proposes a new definition of TRD and introduces the definition of depression resistant to monoamine transporter inhibitors, as we believe that the type of therapy and its pharmacodynamic/receptor profile should be considered when determining response and resistance to treatment. Despite the availability of multiple treatments for depression, including non-pharmacologic ones, many patients with TRD do not achieve a meaningful response (6). In this context, intranasal esketamine arises as a promising and novel treatment option that improves neuroplasticity and brain connectivity, both highly impacted in depression (44). The mechanism of action of intranasal esketamine differs from that of conventional AD, which work mainly through increasing the levels of monoamines in the intrasynaptic cleft (19, 21, 22), and, thus, displays a different clinical performance (both in time of onset and size effect) to that observed with monoaminergic-targeting AD. To facilitate its use in clinical practice, we proposed a treatment algorithm for the use of intranasal esketamine in Spain as a third-line option. Notably, the algorithm highlights that response to any treatment should be evaluated 4–6 weeks after initiation to avoid therapeutic inertia, as this will ensure that a different treatment strategy is considered if a partial or lack of response is detected.

The main strength of this study is the development of the first consensus on TRD and intranasal esketamine use in Spain, driven by experts who considered the socio-cultural context and particularities of its healthcare system. A structured methodology, the nominal group technique, was used to seek consensus and ensured that all experts weighed in on each topic, and that the consensus statements were revised and modified, if needed, by all member of the scientific committee. A limitation of this study is that the non-systematic literature review, which may have resulted in some relevant sources being missed.

## 5 Conclusions

In this study, a committee of psychiatrists from Spain who are experts in TRD reviewed the literature and provided guidance on the definitions of TRD and partial or lack of response, highlighted causes and consequences of therapeutic inertia, and discussed the role of intranasal esketamine in the treatment landscape of TRD, proposing an algorithm for its use in Spain. Given the heterogeneity in the definition of TRD and its treatment, we aim for our recommendations and consensus statements to facilitate management of this patient population.

## Data Availability

The raw data supporting the conclusions of this article will be made available by the authors, without undue reservation.
